# Supplementary material for: Tungsten Diselenide Nanoparticles Produced via Femtosecond Ablation for SERS and Theranostics Applications
Source: Nanomaterials (Basel). 2024 Dec 24;15(1):4. doi: 10.3390/nano15010004 (PMC11721788; doi:10.3390/nano15010004)
Supplement: Supplementary file 1 [file nanomaterials-15-00004-s001.zip › nanomaterials-3353080-supplementary.pdf]

Supporting Information for

Tungsten Diselenide Nanoparticles Produced via Femtosecond Ablation for SERS and Theranostics Applications

Andrei Ushkov[1], Dmitry Dyubo[1], Nadezhda Belozerova[1,2], Ivan Kazantsev[3], Dmitry Yakubovsky[1], Alexander Syuy[1,3], Gleb V. Tikhonowski[3,4], Daniil Tselikov[4,1], Ilya Martynov[1], Georgy Ermolaev[3], Dmitriy Grudinin[3], Alexander Melentev[1], Anton A. Popov[4], Alexander Chernov[1], Alexey D. Bolshakov[1,5,6,7], Andrey A. Vyshnevyy[1,3], Aleksey Arsenin[1,3,7], Andrei V. Kabashin[8], Gleb I. Tselikov[3], and Valentyn Volkov[3,*]

[1]Moscow Center for Advanced Studies, Kulakova str. 20, Moscow, Russia
[2]Frank Laboratory of Neutron Physics, Joint Institute for Nuclear Research, 141980, Dubna, Russia
[3]Emerging Technologies Research Center, XPANCEO, Internet City, Emmay Tower, Dubai, United Arab Emirates
[4]MEPhI, Institute of Engineering Physics for Biomedicine (PhysBio), 115409, Moscow, Russia
[5]Faculty of Physics, St Petersburg State University, Universitetskaya Emb. 7-9, 199034 St Petersburg, Russia
[6]Alferov University, Khlopina 8/3, St. Petersburg 194021, Russia
[7]Laboratory of Advanced Functional Materials, Yerevan State University, Yerevan, 0025 Armenia
[8]Aix-Marseille University, CNRS, LP3, 13288, Marseille, France
[*]Correspondence: vsv@xpanceo.com; Tel.: +971 52 631 9538

This file includes:

Supporting text

Supplementary figures S1 to S5

Supplementary Table S1

Supplementary equations S1 to S10

Supplementary Note S1.

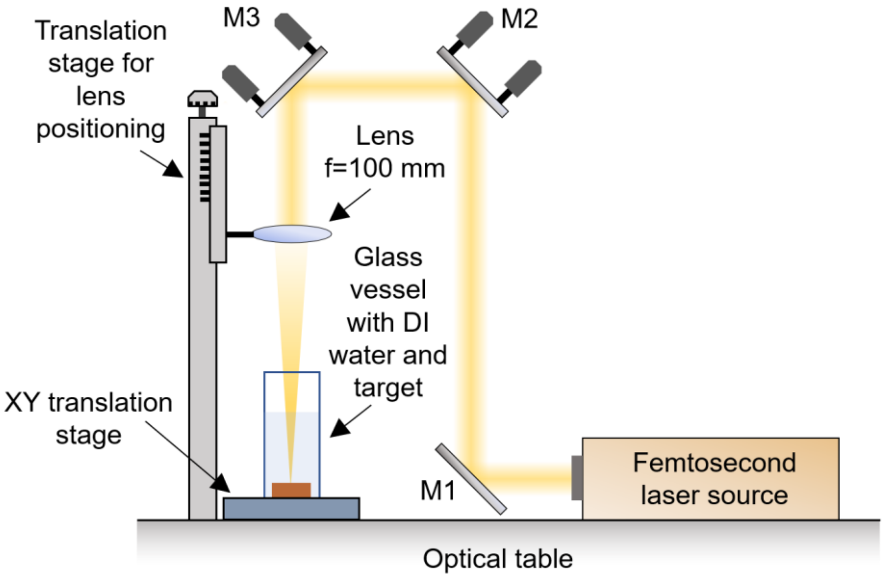

Supplementary Figure S1. The scheme of the experimental femtosecond ablation setup. Mirror M1 is fixed, whereas mirrors M2 and M3 have kinematic mounts for optical path alignment.

Supplementary Note S2.

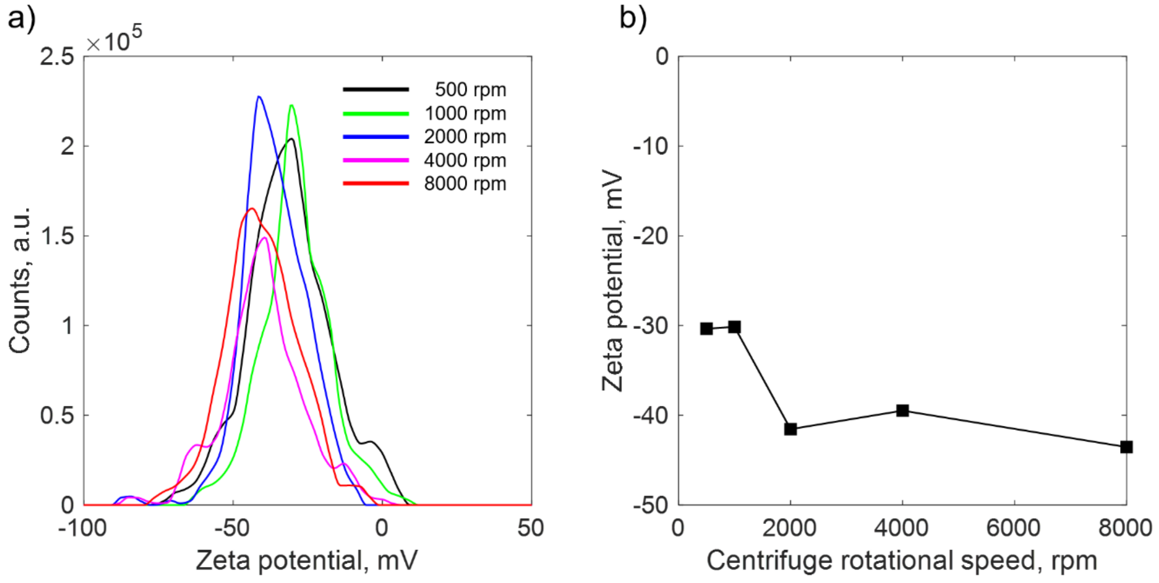

Supplementary Figure S2. Measured zeta potential of WSe_2 colloidal NPs. a) Zeta potential distribution in every colloid; b) Variations of the most probable zeta potential with centrifuge rotational speed.

Supplementary Note S3.

SAED (Fig.2d) demonstrates a polycrystalline nature of synthesized WSe_2 NPs. SAED radial distribution (white spectrum in Fig.2d) reveals diffraction rings positions. The information about corresponding interplanar spacings are summarized in Supplementary Table S1.

Supplementary Table S1. Iinterplanar spacings d_{exp}, determined from SAED pattern of WSe_2 NPs, and calculated d_{hkl} distances between consecutive crystallographic planes with Miller indices hkl.

**WSe_2**			
Ring #	d_{exp} (Å)	hkl	d_{hkl} (Å)
1	3.31	004	3.24
2	2.9	100	2.84
3	2.23	006	2.16
4	2.03	104	2.13
5	1.74	106	1.72
6	1.66	110	1.64

Supplementary Note S4.

Temperature-dependent Raman signal unblocks the possibility to measure the sample temperature, which we employed in the main text (see Fig.4b). Here we compare our data, obtained using LINKAM

temperature control stage for Raman setup, with the data from the literature. The first-order temperature coefficients χ_1 are obtained by the linear fitting of data using the Eq.1 (see the main text).

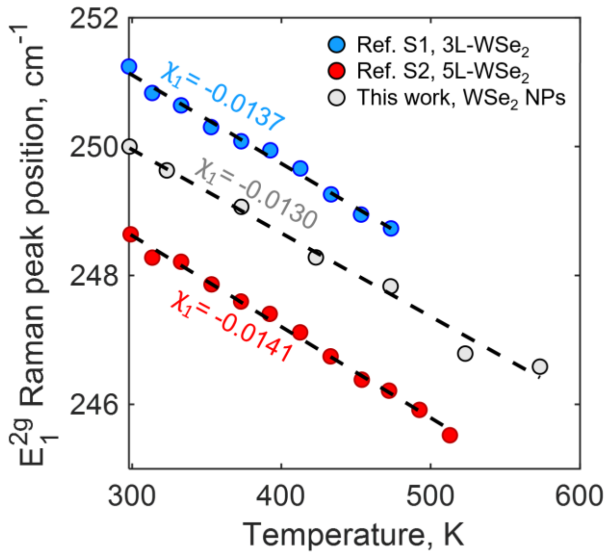

Supplementary Figure S3. Comparison of experimental first-order temperature coefficients χ_1 from the literature and in the present work. Dashed lines denote linear fitting of experimental data (color circles).

Supplementary Note S5.

As it is shown in Fig.4e, the photoheating experiments were performed for water colloids of Si and WSe_2 NPs with average NP diameters $\langle D_{Si} \rangle$=55 nm and $\langle D_{WSe2} \rangle$=48 nm, respectively. Mie theory allows calculating of extinction and absorption cross sections for both colloids, see Supplementary Figure S4.

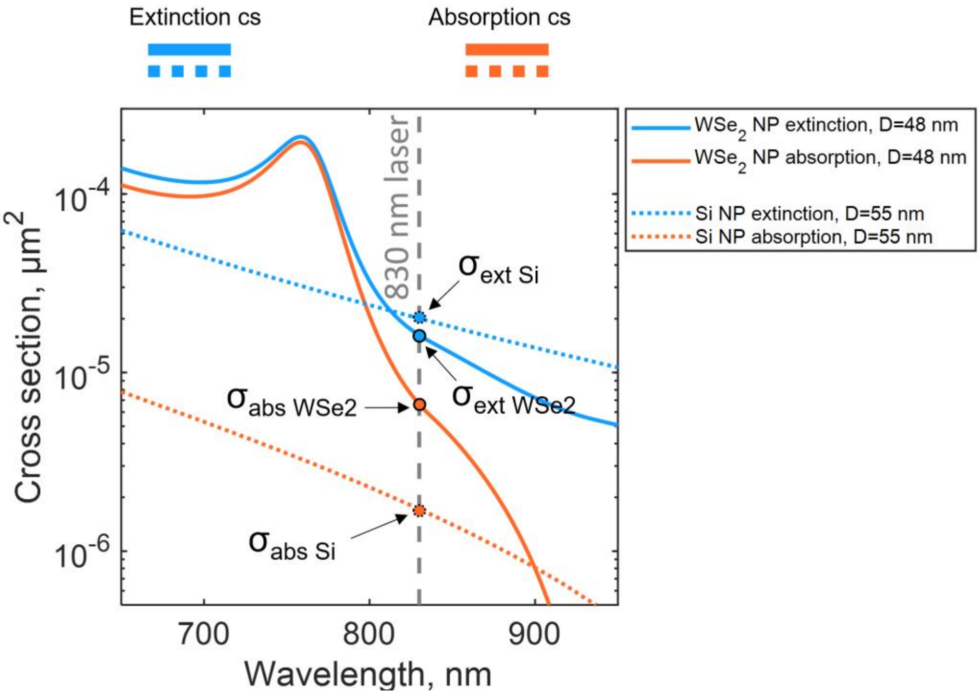

Supplementary Figure S4. Comparison of theoretical extinction and absorption curves of WSe_2 and Si water colloids, calculated via Mie theory. The vertical dashed line corresponds to the 830 nm laser diode line used for photoheating experiments.

At 830 nm cross-sections values are: $\sigma_{ext\ Si}$=2.00958·10^{-5} µm^2, $\sigma_{abs\ Si}$=1.71907·10^{-6} µm^2, $\sigma_{ext\ WSe2}$= 1.61653·10^{-5} µm^2, $\sigma_{abs\ WSe2}$=6.64807·10^{-6} µm^2.

Colloids of Si and WSe_2 in the main text were normalized at 830 nm in order to compare the phototermal conversion efficiency. A theoretical analog of this normalization is a comparison of relative colloidal absorptions (normalized to the total extinction) instead of their absolute values:

$$\frac{\sigma_{abs\ WSe2}}{\sigma_{ext\ WSe2}} \approx 4.8\frac{\sigma_{abs\ Si}}{\sigma_{ext\ Si}}$$

Thus, at the same extinction the colloidal solution of WSe_2 absorbs more than 4 times more 830 nm irradiation, than the solution of Si, what is approved experimentally in Fig.4e.

Supplementary Note S6.

To calculate the photothermal conversion efficiency η we follow an approach described in [S3]. For relatively low heatings the master equation for the thermogram *ΔT(t)* can be written as:

$$C \cdot \Delta\dot{T} = P - L \cdot \Delta T(t), \quad \text{(Eq. S1)}$$

where C is the heat capacity ($C \cong m_{water}C_{water}$), L is the linear losses coefficient, P is the optical power absorbed by NPs, dot above the variable denotes the time derivative. In photoheating experiments we used 1 ml water colloids of NPs.

Under the laser irradiation P>0 the solution of Eq. S1 is a “heating curve”:

$$\Delta T(t) = \xi + B \cdot \exp(-t/\tau), \quad \text{(Eq. S2)}$$

where $\xi = P/L$, $\tau = C/L$, ξ is defined from initial conditions.

In the absence of laser irradiation (P=0) the solution of Eq. S1 is a “cooling curve”:

$$\Delta T(t) = \Delta T_{max} \cdot \exp\left(-\frac{t}{\tau}\right), \quad \text{(Eq. S3)}$$

where ΔT_{max} is the colloid temperature at the moment when the laser was turned off, see Supplementary Figure S5a.

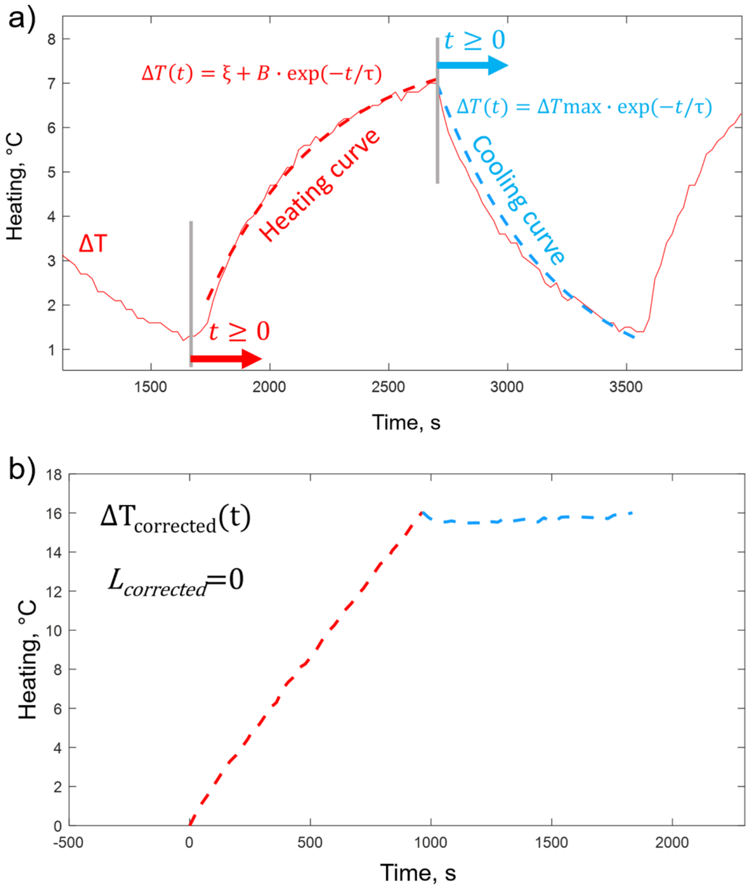

Supplementary Figure S5. a) Definitions of “heating” and “cooling” curves and their fitting using Eqs. S2,S3; b) The same thermogram as in a), re-calculated in the adiabatic representation via Eq. S4.

After some math it is possible to prove that the following change of coordinates:

$$\Delta T_{corrected}(t) = \int \left(\dot{\Delta T} + \frac{\Delta T}{\tau}\right) dt \quad \text{(Eq. S4)}$$

allows to exclude the losses coefficient L from the system, thus making it adiabatic ($L_{corrected}$=0):

$$C \cdot \dot{\Delta T}_{corrected} = P, \quad \text{(Eq. S5)}$$

see Supplementary Figure S5b.

In adiabatic representation, the photothermal conversion efficiency η can be obtained as

$$\eta = C\,\frac{\dot{\Delta T}_{corrected} - \dot{\Delta T}_{corrected\ water}}{I_0(1-Tr)} \quad \text{(Eq. S6)}$$

where $\dot{\Delta T}_{corrected\ water}$ is calculated for the pure water, I_0 is the laser beam power after it passes the pure water cuvette and $I_0 \cdot Tr$ is the power after the actual water colloid of NPs.

Taking into account that P is

$$P = C \cdot \left(\dot{\Delta T}_{corrected} - \dot{\Delta T}_{corrected\,water}\right), \qquad \text{(Eq. S7)}$$

colloidal extinction is

$$I_{ext} = 1 - Tr \qquad \text{(Eq. S8)}$$

colloidal absorption is

$$I_{abs} = P/I_0 \qquad \text{(Eq. S9)}$$

the colloidal absorption curve in Fig.4f can be obtained from the photoheating experiment as

$$I_{abs} = \eta \cdot I_{ext} \qquad \text{(Eq. S10)}$$

References

S1. Easy, Elham, et al. "Experimental and computational investigation of layer-dependent thermal conductivities and interfacial thermal conductance of one-to three-layer WSe2." ACS Applied Materials & Interfaces 13.11 (2021): 13063-13071.

S2. Li, Zhonglin, et al. "Temperature-dependent Raman spectroscopy studies of 1–5-layer WSe2." Nano Research 13.2 (2020): 591-595.

S3. Abu Serea, Esraa Samy, et al. "Enhancement and tunability of plasmonic-magnetic hyperthermia through shape and size control of Au: fe3O4 janus nanoparticles." ACS Applied Nano Materials 6.19 (2023): 18466-18479.
